# Attitudes to Exercise and Diabetes in Young People with Type 1 Diabetes Mellitus: A Qualitative Analysis

**DOI:** 10.1371/journal.pone.0137562

**Published:** 2015-10-14

**Authors:** Kirsty Ryninks, Eileen Sutton, Elizabeth Thomas, Russell Jago, Julian P. H. Shield, Christine P. Burren

**Affiliations:** 1 Psychological Health Service, Bristol Royal Hospital for Children, University Hospitals Bristol NHS Foundation Trust, Upper Maudlin Street, Bristol, BS2 8BJ, United Kingdom; 2 NIHR Biomedical Research Unit in Nutrition, Diet & Lifestyle, University Hospitals Bristol NHS Foundation Trust, Education Centre, Upper Maudlin Street, Bristol, BS2 8AE, United Kingdom; 3 Department of Paediatric Endocrinology and Diabetes, Bristol Royal Hospital for Children, University Hospitals Bristol NHS Foundation Trust, Upper Maudlin St, Bristol BS2 8BJ, United Kingdom; 4 Centre for Exercise, Nutrition and Health Sciences, School for Policy Studies, University of Bristol, 8 Priory Road, Bristol, BS8 1TZ, United Kingdom; 5 School of Clinical Sciences, University of Bristol, 69 St Michael's Hill, Bristol, BS2 8DZ, United Kingdom; University of California, San Francisco, UNITED STATES

## Abstract

**Aims:**

To investigate young people’s attitudes to, and understanding of, physical activity on glycaemic control in Type 1 Diabetes Mellitus.

**Methods:**

Four focus groups with 11–14 and 15–16 year olds were conducted with twelve young people with Type 1 Diabetes, from within a larger study investigating physical activity and fitness. Qualitative analysis of the focus group data was performed using Interpretative Phenomenological Analysis.

**Results:**

Four superordinate themes were identified: Benefits of Exercise, Knowledge and Understanding, Information and Training and “You can do anything”. Young people felt that exercising helped them to manage their diabetes and had a beneficial psychological and physical impact on their bodies. They reported a lack of knowledge and understanding about diabetes among school staff and other young people. The overwhelming sense from young people was that although diabetes impacts upon their lives, with preparation, physical activity can take place as normal.

**Conclusions:**

Whilst young people had an awareness of the physical and psychological benefits of exercise in managing their diabetes, they experienced difficulties at school. Professional support and discussions with young people, giving tailored strategies for managing Type 1 Diabetes during exercise are needed. Healthcare teams should ensure that the support and educational needs of school staff are met. Providing more opportunities to empower young people to take on the responsibility for their Type 1 Diabetes care is merited. Young people felt diabetes did not stop them from participating in activities; it is simply a part of them that needs managing throughout life.

## Introduction

The prevalence of Type 1 Diabetes Mellitus (Type 1 DM) continues to rise, with a current prevalence of 25,000 children and young people in England and Wales [1]. Management is complex to optimise glycaemic control and reduce long-term complications [2]. Healthcare teams advise and empower children and families to monitor blood glucose, consume a healthy diet, increase physical activity, whilst adjusting insulin to match diet and activity variations [3].

Physical activity is recognised as beneficial to all children and young people. Pivovarov *et al* have recently reviewed the additional benefits of physical activity to those with diabetes [4]. Previously, some young people with type 1 diabetes were restricted from organised sport [5]. It had been debated whether exercise is associated with better diabetes control [6] and a recent meta-analysis of exercise interventions to improve control had showed negligible benefit [7]. However, contemporary studies examining activity levels show that self-reported moderate-to-vigorous physical activity is associated with better glycaemic control in young people with diabetes [8]. This is reinforced by accelerometry studies where physical activity is associated with lower HbA1c [9, 10].

Additional barriers to physical activity participation may potentially impact those with diabetes. These barriers could encompass actual changes in glycaemic control due to the exercise or the concern of their occurrence. This concern, such as fear of hypoglycaemia, could arise either in the individual themselves or other parties, e.g. school teachers. Unfortunately, overall levels of exercise remain disappointingly low. We have recently reported that children with Type 1 DM [10] engaged in only 28 minutes of moderate-to-vigorous physical activity per day, which compares poorly with the Department of Health (UK), national recommendations of 60 minutes physical activity per day for all children. Thus, there is a need to identify key mediators to target to facilitate increased physical activity [11], and to understand whether any diabetes specific factors contribute to suboptimal activity levels. Our study examined factors that facilitate or deter physical activity in young people with Type 1 DM, including enjoyment of physical activity, confidence in managing diabetes and possible hypoglycaemic fears influencing attitudes towards exercise.

## Methods

Qualitative design was employed to gain content rich information. Focus groups were selected as the best method for exploring views and experiences as they provide opportunity for group interaction, encouraging participants to explore individual and shared perspectives [12,13].

### Study design and Participants

This qualitative project was conducted in a subgroup of a larger study of physical activity and fitness in young people with Type 1 DM. Patients were recruited from the Bristol and Weston Paediatric Diabetes Service: of 422 patients, 240 aged 8–16 years, exclusion criteria included current hypoglycaemia unawareness and co-morbidities such as severe asthma or physical disability, leaving 215 eligible young people (60 of whom participated in the main study) [10].

Young people aged 11 to 16 years with Type 1 DM who had consented to being approached about a focus group were telephoned by the research nurse. Those confirming interest received a focus group invitation letter. Focus groups were conducted by a research assistant, research nurse and medical student, independent of the clinical team. This study including consent procedure was approved by the Southmead Ethics Committee (REC ref. 07 ⁄H0102⁄73). Written informed consent was obtained from parent or guardian for all participants and recorded through filing in their hospital notes.

Four focus groups with 11–14 and 15–16 year olds with Type 1 DM were conducted with twelve young people: mean age 14.5 years (SD = 1.5), (Table 1: demographic information). Focus group participants (n = 12) had similar glycaemic control in 2010 to the main study group (n = 60): median HbA1c 66 mmol/mol (8.3%) and mean 68 mmol/mol (8.4%) respectively. There was also no significant difference in diabetes duration between the focus group participants and the main study group. In the main study [10], Moderate-to-Vigorous-Physical Activity (MVPA) on accelerometer testing was found to associated with better glycaemic control. There was no significant difference *(p 0*.*09)* in MVPA between the focus group participants (mean 20.6 minutes per day) and the others from the main study group (mean 30.4 minutes per day) Table 1 also shows median HbA1c at the time of focus group in 2013, and changes in insulin regimen types.

**Table 1:** Specific descriptive data in focus group participants (n = 12).

	*n = (%)*
Gender	
Male	8 (67%)
Female	4 (33%)
Ethnicity	
White British	9 (75%)
Polish / White—Any Other	1 (8.3%)
Black British Caribbean	1 (8.3%)
Mixed–Any Other	1 (8.3%)
Parental Education	
None	0 (0%)
NVQ L1	1 (9.1%)
GCSE or equivalent	4 (36.4%)
A-Level	3 (27.3%)
Degree	2 (18.2%)
Higher degree	1 (9.1%)
Insulin Regimen	BD, MDI, Pump
2010	1 (8.3%), 6 (50%), 5 (42%)
2013	0 (0%), 4 (33%), 8 (67%)
HbA1c	Median
2010	67 mmol/mol (8.3%)
2013	65 mmol/mol (8.1%)

Abbreviations: NVQ non-vocational qualification, GCSE General Certificate of Secondary School Education, BD twice a day, MDI Multiple Daily Injections

All focus groups were audio recorded and lasted 30 to 45 minutes. The groups were semi-structured and facilitated aided by a topic guide, developed following literature review. Open ended questions were used, such as: “*Do you think exercise helps people with diabetes*?” The semi-structured format enabled the young people’s own experiences to direct the discussion. The focus groups examined factors that facilitate or deter physical activity in young people with Type 1 DM, including enjoyment of physical activity, physical activity preferences, confidence in managing diabetes and possible hypoglycaemic fears influencing attitudes towards exercise.

### Data collection and analysis

Data were analysed using Interpretative Phenomenological Analysis (IPA), used previously to explore young people’s experiences of Type 1 DM [14,15], this method of analysis recognises that personal, societal, cultural and familial perspectives strongly influence experience of the world [16]. IPA enables construction of a framework to classify and organise information according to key themes, concepts and emergent categories. IPA is concerned with individuals’ subjective reports, rather than formulating an objective account and is therefore considered phenomenological and a dynamic process. [17].

Focus groups were transcribed by KR (research assistant) and all patient identifiable details were removed. Transcripts were checked for accuracy and pseudonyms inserted. Focus group data were analysed manually by the research assistant for recurrent themes, to classify and code information according to key themes, concepts and emergent categories. Initial codes and notes of association, summarised experiences that young people described. Key phrases from young people were used to label themes and a preliminary themes list was compiled and examined to coherently group together and cluster by shared meaning. This process was followed for transcripts from each focus group. Themes were refined and clustered to develop super-ordinate and master themes, which were checked against the original transcripts to ensure shared meaning was maintained across analyses. Triangulation with a senior researcher in qualitative analysis (ES) achieved credibility checks.

## Results

Four superordinate themes and nine subordinate themes were developed (see Table 2) following analysis of focus group transcripts. These themes are discussed, accompanied by selected illustrative quotations (additional quotes in Table 2).

**Table 2:** Superordinate and subordinate themes from focus groups.

SUPERORDINATE THEMES	SUBORDINATE THEMES	Selected illustrative quotations
1 Benefits of Exercise	1.1 Managing Diabetes	*Exercise kind of helps lower your sugar level*. *If it’s high then you just go outside for a run and then it goes back to normal again (Edward*, *15y)*
	1.2 Physical and Psychological impact	*When you’re doing exercise you know you’re helping your body as well as yourself (Sam*, *15y)*. *If you have a pump on it might not be great to do stuff that’s too dangerous*, *in case it gets damaged (Jake*, *15y)*
2 Knowledge and Understanding	2.1 Schools	*When I was at school and P*.*E*. *teachers they like kept pestering me if I’m okay*, *it’s like I’ll stop if I’m not (Miles*, *16y)*
	2.2 Young people	*They think it kind of like restricts what you can actually do (Ben*, *14y)*. *My friends are very supportive of my diabetes and they understand…some people are a bit kind of cautious and um of it and they’ll ask questions and things*. *But um you know when they know that I’m diabetic they’re very understanding (Ellie*, *12y)*
3 Information and Training	3.1 School	*I think like actually showing the P*.*E*. *teachers what to do if something happens so they don’t hold back that student just because they have diabetes and it will encourage them to go further (Robert*, *13y)*
	3.2 Healthcare Team	*I don’t think I’ve ever talked about exercise in the clinic*, *unless I’ve specifically gone here to talk about exercise… They don’t really ask you about sports and they just–they might query why you might be low here or high there*, *but not directly about sports*. *(Robert*, *13y)*
4 You can do anything	4.1 Preparation is key	*You can eat whatever you want*, *you can do whatever you want*.* *.* *. *But we just have to do a tiny bit more work on our levels (Jonah*, *13y)*
	4.2 Family Support	*I think my family’s quite encouraging… if you believe in yourself*, *you know you can make it and it doesn’t really matter*. *You can do anything (Robert*, *13y)*
	4.3 Life goes on	*It motivates me that I know it can’t hold me back*. *Even if you have got it*, *it’s part of life now (Miles*, *16y)*

### Benefits of exercise

Young people spoke at length about the physical and psychological impact of exercise on their bodies, as well as the perceived benefits of exercise in managing their diabetes. They reported exercising “*keeps your blood sugar at good rates*” (Hannah, 13y) and ‘‘*It helps to sort of control*. *I don’t really know why but I felt that um*, *if I’m doing more exercise um I can normally keep my levels at a more consistent rate*.*” (Jake*, *15y)*

They described particular activities where they noticed a positive difference in blood glucose following exercise:

*Walking is really good for my blood sugars my mum says*. *I went on a walking weekend with Guides and my blood sugars were really good all the time I was doing that*. *So that’s good for my blood sugars (Ellie*, *12y)*

As well as exercise helping them to manage their diabetes, five young people talked about the positive psychological and physical impact that exercise has on their bodies:

*I feel better for it but that’s probably just ‘cause I’m enjoying my sport (Andrew*, *16y)*

*it keeps you fit and um as well um if you um eat properly as well*, *then like I said it keeps you fit and nice and healthy (Ellie*, *12y)*

Two young people also described the impact of having a pump and the effect on their physical ability to exercise:

*I find swimming is one of the most difficult ‘cause the pump’s not on you and so I can only do like half an hour/45 minutes before I start feeling…high or really low*. *So that’s a bit annoying…it’s the only thing that I would say with the pump*, *it’s a bit of a drawback (Miles*, *16y)*

Exercise was reported to be beneficial by eight young people in helping them to manage their diabetes and feeling physically and psychologically well, although two young people reported that their ability to engage in exercise such as swimming was limited by having a pump.

### Knowledge and understanding

Knowledge and understanding was another key theme. Young people reported experiencing difficulties at school, with teachers being over-vigilant and other young people misunderstanding the impact of diabetes on their life. Although it was sometimes useful when teachers checked on them in class, at other times young people found it frustrating being constantly asked if they were okay:

*My biology teacher often checks if I’m okay*, *more than most of my teachers probably just cause she understands it more…(frustrating) if I’m fine*, *but if I’m not then it’s useful (Jake*, *15y)*.

Three young people reported experiencing particular difficulties with their P.E. teachers, who often held them back from taking part in certain activities fearing what might happen:

*I’d usually be kept on the side for the start of the season cause they’d say*, *‘Oh we’ll only bring him on if we need to bring him on cause we don’t want to risk this and that*,*’ and even if I said to them I don’t need to be kept off*, *I can do this*, *they’d say we don’t want to risk anything*. *It was only towards the end of the season when they’d bring me on if someone else was taken off and they saw I could actually do well*, *that they kept me on for the whole game…I*, *personally I think it*, *they were scared that if something did go wrong I’d end up hypo and I went like really bad and fell over or something they would panic and not know what to do*. *So they would try to keep it as far as away from that possibility of happening as much as they possibly could (Robert*, *13y)*.

These findings highlight a need to educate school staff in hypoglycaemia prevention and management. It is interesting to reflect that this ‘fear of hypoglycaemia risk’ that school staff expressed, was not a theme that emerged as a concern of the young people themselves. Four young people also reported that their peers thought diabetes was a more serious condition than they personally believed it to be and explained the consequent negative impact on their confidence in participating in physical activity:

*Sometimes it’s people*, *other people who are*, *who are fit… They create a mental block which sort of*, *when they start making like sarcastic comments with stuff like ‘oh you can’t do this*, *you’re diabetic’*, *and it puts that mental block in your brain*, *which makes you think I can’t do this sport anymore and that makes you not do enough exercise (Robert*, *13y)*.

Although some young people reported experiencing difficulties with their peers, others found it helpful for their friends to know about their diabetes and felt more supported about disclosing their condition to those around them:

*If we didn’t tell anyone they wouldn’t know what to do (Jonah*, *13y)*.

*I only sort of tell people actually when they need to know… So it’s like my friends know about it and they can help with it and don’t really need to make like other people like in your different classes know about it (Lucy*, *13y)*.

Whilst four young people found it helpful for their peers and school teachers to know about their diabetes, five young people reported experiencing significant difficulties with their peers and teachers being overly-cautious, misunderstanding the limitations of diabetes and the effect it has on their ability to participate in exercise.

### Information and training

As well as the difficulties reported above, young people were proactive in suggesting ways to improve the knowledge and understanding of those around them and spoke more generally about living with Type 1 DM and the type of support they would like from health professionals. Developing resources for PE teachers that explain diabetes and the effect of various activities would be helpful, particularly for those newly diagnosed:

*I think maybe people who are like newly diabetic um*, *you know they*, *maybe they could have a leaflet for like if they go back to school and they don’t know what to do um in P*.*E*. *or something like that*, *then that could be useful to them (Ellie*, *12y)*.

Such resources would be particularly helpful for young people, as participants reported that they were often the ones who had to explain their condition to teachers at school:

*At schools like maybe telling the P*.*E*. *teacher ‘cause it’s a bit annoying for the child that they’ve just got it*, *to try and tell a P*.*E*. *teacher ‘cause you don’t really feel comfortable*, *I never did*. *Saying to an adult ‘oh this is what I need to have’ (Miles*, *16y)*.

Greater input from their healthcare team giving more information about how to exercise with diabetes was also something that four young people reported would be beneficial:

*Maybe when you’re first starting*, *‘cause I got er the pump 7 or 8 years ago now and it was quite er*, *very new then and you didn’t really know how to manage it with certain things like*, *like it was of those where I hadn’t always noticed about taking if off for exercise and stuff like that*. *So maybe some explanation on that (Edward*, *15y)*.

*You could also like just give them like a recommendation ‘cause it’d just be easier for them*. *Less insulin and then it would be just like less amount works (Jonah*, *13y)*.

Three young people felt they would like to be more involved in discussions around their healthcare and the management of their diabetes:

*I think maybe they should ask me a few more questions because*, *you know*, *I’ve kind of got to deal with it you know*, *every day in school and things*. *(Ellie*, *12y)*

Providing information and resources about diabetes for schools, and increasing discussions about exercise and managing diabetes in clinic consultations with their healthcare team were some suggestions young people provided to increase the knowledge and understanding of diabetes in those around them. Teachers may be less cautious and more supportive of physical activity if they are better informed.

### “You can do anything”

Another key theme was the concept that “You can do anything”. Preparation was identified as key to participating in normal activities, such as making “*sure you have enough snacks that keep your sugars up if it goes low*” (Hannah, 13y). Five young people reported:

*If you’re prepared*, *you can really do kinda anything*. *If you’re not prepared*, *I’m not prepared to even go into an activity (Miles*, *16y)*

Family members played a key role in young people feeling supported in managing their diabetes and encouraging them that they can do anything:

*My mum was interested about sport and diabetes so she went on the internet…there were four people in the Olympics that had diabetes…I was interested that (diabetes) holds you back a bit but not too much (Miles*, *16y)*.

The overwhelming sense from young people with diabetes consulted in this study was that life goes on:

*You control diabetes rather than it controlling you*. *So if it controls you then yes it will stop you doing some certain things ‘cause you’re too unhealthy and you know you’re not feeling right*. *But if you control it well then you know you’re just normal*, *you’re a normal person so you can do it like anyone else can (Sam*, *15y)*

For the twelve young people involved in the four focus groups, although diabetes impacts upon their lives, with preparation, “*diabetics can do pretty much everything that non-diabetics can do*” (Ellie, 12y).

## Discussion

The data reported in this paper show that young people with Type 1 felt that exercising helped them manage their diabetes, and had a beneficial psychological and physical impact on their bodies. Interestingly, the children themselves did not bring up risk of hypoglycaemia as a major concern mirroring the findings in a recent study of adults with type 1 diabetes in relation to physical activity [18]. Nevertheless, they reported a lack of knowledge and understanding among school staff (including PE teachers) and other young people about their condition, and highlighted the need for information provision and training for schools, as well as greater input from their healthcare team on how to manage their diabetes when they exercise. They reported that although diabetes impacts upon their lives, with preparation and support from family and friends, life can go on as normal.

The results highlight a range of issues particularly relevant to health professionals working with young people with Type 1 DM. Young people were aware of the health benefits of exercise in managing their diabetes. Recent studies indicate that moderate-to-vigorous physical activity is associated with better glycaemic control in young people with Type 1 DM [9,10]. Previous research has also shown that in addition to exercise having beneficial effects on cardiovascular risk factors, weight loss and physical conditioning, physical activity is associated with positive mental well-being [19]. Psychological benefits include improved self-esteem and an enhanced quality of life [20]. It is important for health care teams to highlight the importance of engaging in physical activity.

Two young people described the impact of having a pump, including limitations on engaging in activities such as swimming. Insulin pump therapy enables basal insulin adjustment for exercise. However, difficulty in managing glucose levels when the pump was disconnected for swimming was reported. Professional input giving tailored strategies for managing these situations should be provided. These may include eating certain snacks and adjusting insulin levels before or after exercise. The International Society for Pediatric and Adolescent Diabetes (ISPAD) provides recommendations for managing Type 1 DM during school activities [21].

Policy recommendations and guidelines recognise that schools are an important environment for increasing physical activity [22], nevertheless earlier research reported that teachers were apprehensive about possible negative effects of hypoglycaemia, tending to over treat hypoglycaemic episodes due to lack of understanding about diabetes [23], and that a lack of understanding among teachers was common [24,25]. Disappointingly our study suggests minimal progress in restriction on participation by school staff since earlier reports. Young people in our study spoke at length about the difficulties they experienced at school, with over vigilant teachers and peers misunderstanding how diabetes affects their life. Similarly, another recent study reported that only 75% of staff thought you could exercise if you had diabetes [26]. Both illustrate unresolved barriers to delivering policies and guidelines which need to be addressed.

Our data shows that young people with Type 1 DM continue to experience significant difficulties at school. The level of support received in school varies greatly; key barriers to the provision of support in schools include lack of training and concerns over liability [27]. Nabors et al [28] identified the difficulties encountered by young people wanting to participate in after school clubs, including lack of access to hypoglycaemia treatments, sports coaches having insufficient knowledge of diabetes to provide adequate support and to recognise and treat hypoglycaemic episodes. From September 2014, schools were required by law to ensure all children with a medical condition receivethe care and support they require to participate fully in school life [29] and health care professionals must ensure that the support and education that school staff need is available.

Tahirovic and Toromanovic [30] reported that although children with Type 1 DM may not have appropriate diabetes care in schools, school staff are generally keen to solve this problem. Capitalising on this willingness, the development of resources for P.E. teachers, explaining what Type 1 DM is and the effect of various activities, would be particularly helpful. Young people also reported that it would be helpful to have greater input from their healthcare team on how to manage their Type 1 DM when they exercise, and they wanted to be provided with more opportunities to take on the responsibility of their diabetes care. One young person described how she often felt ignored in her clinic appointments, with the adults tending to talk to one another as if she wasn’t there. It is important that health professionals involve young people in the management of their diabetes.

Management of Type 1 DM is complex for young people due to the need to integrate lifestyle modifications (eating and exercising) and daily medical tasks (blood glucose monitoring and insulin injections) into everyday life. Preparation was reported to be a key enabler to young people with Type 1 DM being able to take part in normal activities, with young people explaining that they are able to do what they want, as long as they work to stabilise their levels. Family members also played an important role in young people feeling supported in managing their diabetes. Adolescents with Type 1 DM belonging to supportive families have been found to have better control of their condition [31]. However, although familial support is a necessary aspect of managing diabetes, it is not sufficient to ensure adequate disease management. Participants described how they found it helpful for their friends to know about their diabetes, confiding in them and sharing information about their diagnosis and medical regimen. Diabetes management occurs across multiple settings, including at home and at school, therefore support from family as well as from peers plays an integral role in encouraging and providing support for young people with Type 1 DM.

The overwhelming sense from the young people consulted was that although Type 1 DM impacts upon their lives, life *can* go on as normal and you *can* do anything. Participants did not feel that their condition held them back. This finding was in contrast to research by Graue et al [32], who reported that when compared with healthy adolescents, adolescents with diabetes were more worried, perceived a greater impact of diabetes on daily life and had a lower life satisfaction. The feelings of young people with Type 1 DM in this study are perhaps best summarised by Andrew (16y):

*“Diabetes isn’t the something to stop you doing something*. *It’s just something you have to carry with you to the finish line*.*”*

### Strengths and limitations

Qualitative studies often have small sample sizes which may limit generalisability. However, qualititative research produces detailed information, giving insights that cannot be realised by quantitative research. We acknowledge our small sample size (n = 12); but each group provided detailed and unique description of their experiences, which remain relevant as giving us insight into the thinking processes of young people with diabetes. The emergent themes suggest important areas to explore further in larger populations and amongst school sports teachers.

A few potential limitations exist. Participants appeared well-motivated and keen to talk about their Type 1 DM and exercise and had supportive families. It may be that young people deterred by exercise might not have wanted to participate in focus groups for fear of feeling vulnerable. Furthermore, focus group participants were a subset from the initial larger study with 28% recruitment. So, more sedentary individuals may have been less willing to enrol in that initial study, skewing the range of views expressed in focus groups.

In conclusion, this study adds to current literature on young people’s attitudes to, and understanding of, physical activity on glycaemic control in Type 1 DM, revealing that young people demonstrate awareness of the physical and psychological benefits of exercise in managing their diabetes. However young people also report experiencing significant difficulties at school, highlighting the need for greater consistency in the implementation of school-related policies and practices. Thus our findings draw attention to the need for improved information provision and training to support young people, parents and teachers in schools, as well as greater input for young people from their healthcare team on how to manage their diabetes when they exercise. Preparation and support from family and friends played a key role in young people’s daily management of their Type 1 DM. Moreover, young people emphasised the notion that with appropriate support “*You can do anything*”; diabetes does not have to stop you from living a normal life. These findings are relevant to health professionals, policy makers and health service managers involved in planning and providing paediatric diabetes care.
